# A Comparative Study of Stone Re-Treatment after Lithotripsy

**DOI:** 10.3390/life12122130

**Published:** 2022-12-16

**Authors:** Yueh-Er Chiou, Chi-Hsiang Chung, Wu-Chien Chien, Pei-Kwei Tsay, Hung-Cheng Kan, Wen-Hui Weng

**Affiliations:** 1 Department of Nursing, College of Medicine, Fu Jen Catholic University, New Taipei City 242, Taiwan; 2 Department of Medical Research, Tri-Service General Hospital, National Defense Medical Center, Taipei City 114, Taiwan; 3 School of Public Health, National Defense Medical Center, Taipei City 114, Taiwan; 4 Taiwanese Injury Prevention and Safety Promotion Association, Taipei City 114, Taiwan; 5 Department of Public Health and Center of Biostatistics, College of Medicine, Chang Gung University, Taoyuan 333, Taiwan; 6 Division of Urology, Department of Surgery, Chang Gung Memorial Hospital, Taoyuan 33305, Taiwan; 7 Department of Chemical Engineering and Biotechnology and Graduate Institute of Biochemical and Biomedical Engineering, National Taipei University of Technology, Taipei City 106, Taiwan

**Keywords:** urolithiasis, re-treatment, lithotripsy

## Abstract

The high recurrence rate has always been a problem associated with urolithiasis. This study aimed to explore the effectiveness of single interventions, combined therapies, and surgical and nonsurgical interventions. Herein, three lithotripsy procedures—extracorporeal shock wave lithotripsy (ESWL), percutaneous nephrolithotomy (PCNL), and ureteroscopic lithotripsy (URSL)—were assessed and a retrospective cohort was selected in order to further analyze the association with several risk factors. Firstly, a population-based cohort from the Taiwan National Health Insurance Research Database (NHIRD) from 1997 to 2010 was selected. In this study, 350 lithotripsy patients who underwent re-treatment were followed up for at least six years to compare re-treatment rates, with 1400 patients without any lithotripsy treatment being used as the comparison cohort. A Cox proportional hazards regression model was applied. Our results indicate that the risk of repeat urolithiasis treatment was 1.71-fold higher in patients that received lithotripsy when compared to patients that were not treated with lithotripsy (hazard ratio (HR) 1.71; 95% confidence interval (CI) = 1.427–2.048; *p* < 0.001). Furthermore, a high percentage of repeated treatment was observed in the ESWL group (HR 1.60; 95% CI = 1.292–1.978; *p* < 0.001). Similarly, the PCNL group was also independently associated with a high chance of repeated treatment (HR 2.32; 95% CI = 1.616–3.329; *p* < 0.001). Furthermore, age, season, level of care, and Charlson comorbidities index (CCI) should always be taken into consideration as effect factors that are highly correlated with repeated treatment rates.

## 1. Introduction

The re-treatment of kidney stones occurs within five to ten years in nearly 30% to 50% of patients [1,2]. Stone composition, stone size, patients’ renal anatomy, stone location, heredity, environment, and eating habits all play a role in determining the outcome and operative approach [3,4,5]. As a result of the low level of invasiveness, extracorporeal shock wave lithotripsy (ESWL) is used as the first-line treatment for renal calculi. According to the European Association of Urology (EAU) guidelines, ESWL is considered a treatment option in patients with renal stones (excluding lower polar stones) between 10 and 20 mm in size [6]. The re-treatment rate from 20% to 70%, is dependent on the time period after surgery [7,8]. The multiple treatment sessions required to optimize results is one of the major limitations of ESWL [9,10]. Therefore, alternative treatments should be considered to further reduce re-treatment rates. For example, percutaneous nephrolithotomy (PCNL) is a treatment option that offers the direct removal of stone fragments and offers up to a 95% success rate [11]. In addition to PCNL, ureteroscopic lithotripsy (URSL) allows for the visualization of the entire drainage system of the kidney and can treat stones of up to 20 mm in size. While ESWL and ureteroscopy can be performed under intravenous sedation, PCNL requires general anesthesia, which excludes certain patients. Moreover, the risk of inadvertent organ injuries should always be considered [12,13]. Over 25% of all kidney stone surgeries are now performed using small-scale ureteroscope technology to remove smaller stones [14,15]. Aside from surgery, careful observation is also a strategy, which involves allowing stones to pass naturally without medical intervention [16]. However, it is recommended that stones of size >10 mm be aggressively treated with ESWL, PCNL, URSL, or a combination of methods. As previously mentioned, the decision to intervene depends on understanding the limitations of each treatment modality and carefully considering the patients’ situations [16,17,18].

To date, very few studies have made progress in comparing ESWL, PCNL, and URSL [19,20,21]. We hypothesize that the effect of lithotripsy on the re-treatment of urolithiasis is based on an exposure-response relationship: a process by which the prevalence of the intervention increases as the size of the stone increases. The stone regain rate may be associated with intervention-related morbidities [22]. Therefore, we attempted to use insurance claims data in Taiwan to evaluate the risk of re-treatment after lithotripsy. This was a retrospective cohort study that compared the efficacy of various management options, such as ESWL, PCNL, and URSL, and other possible issues, such as sex, age, season, and level of care. The systemic analysis by the Charlson comorbidities index (CCI, including heart disease, hypertension, respiration disease, dementia, cerebrovascular disease, joint disease, diabetes, sensory impairment), which may be related to an increased risk for urolithiasis, was also considered [8,23,24,25,26,27,28,29,30,31]. The assessment of the factors mentioned above may contribute to the prediction and prevention of urolithiasis re-treatment after lithotripsy.

## 2. Materials and Methods

### 2.1. Data Sources

The database used in this study was based on Taiwan’s National Health Insurance Research Database (NHIRD) as the accuracy and validity of the diagnoses in this database have been demonstrated in several studies [8,28]. From 1995 to June 2009, 97% of the medical providers in Taiwan accepted National Health Insurance (NHI). In addition, more than 99% of the 23 million people in the population were included in the NHI system. The data were compiled by the Bureau of NHI through randomly selecting 1 in every 100 ambulatory care visit records and 1 in every 20 inpatient claims. Therefore, the database used in this study is a representative database provided by the National Health Research Institutes (NHRI) beginning in 2000, when the registry of all NHI enrollees was compiled using a systematic sampling method [17,27,32]. Furthermore, the codes for the medical diagnosis of urolithiasis in the NHIRD were recorded by board-certified urologists using the International Classification of Diseases, 9th Revision, Clinical Modification (ICD-9-CM). Patients and/or the general public were not involved in this study.

### 2.2. Study Design and Sampled Participants

**Figure 1:**
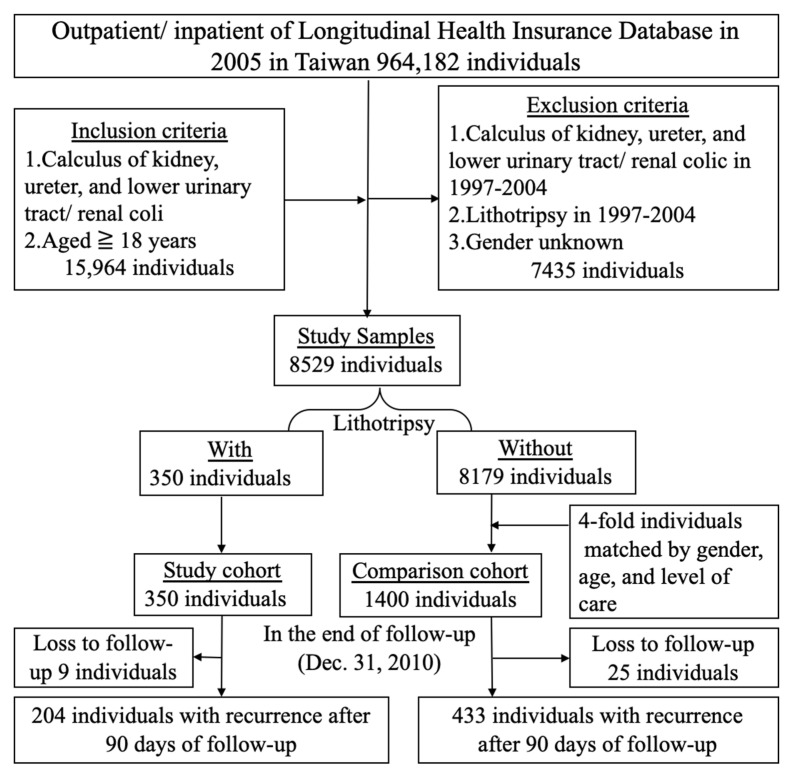
The flowchart of the study sample selection from the National Health Insurance Research Database in Taiwan.

A retrospective matched-cohort design was used for this study. The sample selection is shown in Figure 1. Since the health insurance database was compiled from 1997 to 2010, but the standard for labeling ESWL treatment was unified to the ICD-9 cm in 2000, in order to avoid inconsistencies, only entries after 2000 were used. All the targeted patients were newly diagnosed with urolithiasis between 1 January 2005 and 31 December 2005, regardless of whether they received ESWL (ICD-9 cm OP 98.5, OP 98.51), PCNL (ICD-9 cm OP 55.01, OP 55.03, OP 55.04), or URSL (ICD-9 cm OP 59.95). Patients who were under the age of 18, underwent urolithiasis therapy between 1997 and 2004, or had been treated with lithotripsy before 1 January 2005, were excluded.

Initially, a total of 964,182 participants were identified from the NHIRD for this study. Both inpatients and outpatients from the Longitudinal Health Insurance Database (LHID) were included to represent a valid sample of the NHI program in Taiwan. Among these individuals, 15,964 individuals aged 18 years or older matched the criteria for renal calculus, ureteric calculus, or lower urinary tract/renal colic (calculus of the kidney and ureter: ICD-9 cm 592; calculus of the lower urinary tract: ICD-9 cm 594; and renal colic: ICD-9 cm 788.0; lithotripsy included ESWL, PCNL, and URSL). From this group, 7435 individuals (sex not specified) were excluded due to having experienced urolithiasis between 1997 and 2004, resulting in a group of 8529 individuals, among which 350 had undergone lithotripsy and 8179 had not. We carefully performed 4:1 matching based on the variables of sex, age, and level of care. For the study cohort group of 350 individuals, 1400 individuals who had not undergone the procedure were selected as the comparison cohort group; this group of patients only experienced observation or drug treatment. However, data on 9 patients in the study cohort and 25 patients in the comparison cohort were excluded after follow-up to 31 December 2010 because of a lack of follow-up data for these patients after initial diagnosis or surgery. Re-treatment cases were defined as 90 days after the initial surgery, and were aimed at recruiting patients with relatively high rates of surgical clearance of previous stones; this was following the methodology of previous similar studies such as Abdulla Al-Ansari et al.’s human studies. The purpose was to avoid cases of retreatment due to residual stones after the previous operation [33]. Among these individuals, 204 patients relapsed in the study cohort group (59.82%), and 433 patients in the comparison cohort group experienced re-treatment (31.49%) (Figure 1). A statistical analysis was performed on this modified dataset.

### 2.3. Statistical Criteria

The univariate analysis compared the re-treatment rates following lithotripsy and nonsurgical treatment. In this study, the variable in the study cohort was the type of lithotripsy, which included ESWL, PCNL, and URSL, and the variable in the comparison cohort was nonsurgical treatment. The multivariate analysis included the types of lithotripsy operation, which were grouped as follows: ESWL only, PCNL only, URSL only, ESWL combined with PCNL, ESWL combined with URSL, PCNL combined with URSL, or ESWL combined with PCNL and URSL. The baseline characteristics of the patients in the study cohort group (with lithotripsy) and the comparison cohort group (matched without lithotripsy) were matched 4:1 with the covariates of socio-demography, which included sex, age (18–30, 31–40, 41–50, 51–65, and >65 years old), and level of care (medical center, regional hospital, and local hospital) (Table 1).

In addition, the CCI measures the severity of illnesses, such as hypertension (ICD-9 cm codes 401~405), diabetes mellitus (ICD-9 cm code 250), heart failure (ICD-9 cm code 428), stroke (ICD-9 cm codes 430~438), chronic obstructive pulmonary disease (ICD-9 cm codes 490~496), liver cirrhosis (ICD-9 cm code 571), and Meniere’s disease (ICD-9 cm code 386.0); as long as the patient was diagnosed, these would be recorded. Among environmental factors, the season (spring (March–May), summer (June–August), autumn (September–November), and winter (December–February)) was analyzed. Regarding the type of hospital, the level of care was divided into 3 categories (medical center, regional hospital, and local hospital). Data were collected from the inpatient and outpatient registry files of the NHIRD. In accordance with the regulations, personal identification information was encrypted and unavailable before the data were released for research. The disease diagnoses of individuals were recorded in the outpatient and inpatient data of the LHID according to the standard diagnosis of 2005.

As re-treatment for residual or recurrent stones is a common issue after initial treatment, we defined re-treatment events in the current study as two urolithiasis treatments that were at least 90 days apart. Additionally, to minimize the possibility of bias resulting from this operational definition, we analyzed patient re-treatments. The study cohort groups that presented urolithiasis re-treatment comprised patients re-admitted to the hospital. However, patients with urolithiasis who visited hospitals within 90 days were not considered re-treatment cases as they could have been afflicted by residual stones or could require follow-up treatment.

### 2.4. Data Analysis

All statistical analyses were performed using SPSS version 24 (SPSS Inc., Chicago, IL, USA). χ^2^ and *t*-tests were used to evaluate the distributions of categorical and continuous variables, respectively. Fisher’s exact test was used to examine significant differences between two cohorts for categorical variables with a significance level of *p* < 0.05. The cumulative risks of demographic-specific and comorbidity-specific re-treatment incidence for the study cohort group and the comparison cohort group were compared using a Cox proportional hazards regression model adjusted for potential confounding factors to estimate the hazard ratios (HRs) and 95% confidence intervals (CIs) for the study cohort. Furthermore, the differences in the risk of urolithiasis after lithotripsy between the study cohort and comparison cohort groups were estimated using a Cox model with the log-rank test. A two-tailed *p*-value of <0.05 was considered statistically significant.

## 3. Results

At the conclusion of the follow-up, the characteristics of the patients after lithotripsy, which included patients who underwent ESWL, PCNL, URSL, ESWL + PCNL, ESWL + URSL, PCNL + URSL, and ESWL + PCNL + URSL, were compared with those of the non-lithotripsy comparison cohort group. A total of 341 and 1375 patients were included in the study cohort and comparison cohort, respectively. In the final analysis, 204 out of 341 patients recurred in the study cohort, versus 433 of 1375 patients in the comparison cohort. The mean age of the re-treatment group was 52.07 ± 13.72 years, and that of the subjects without re-treatment was 54.97 ± 14.63 years (Table 2).

### 3.1. Univariate Analysis of the Re-Treatment Rate of Urolithiasis

The re-treatment rate in the group without lithotripsy was 31.5% (433/1375). However, the re-treatment rate was 55.1% (118/214) after undergoing only ESWL; 67.9% (38/56) after receiving only the PCNL procedure; 51.2% (21/41) after receiving only URSL; 88.9% (16/18) after ESWL + PCNL; 85.7% (6/7) after ESWL + URSL; 100% (3/3) after PCNL combined with the URSL procedure; and 100% (2/2) after all three procedures (ESWL + PCNL + URSL) (*p* < 0.001) (Table 2).

**Figure 2:**
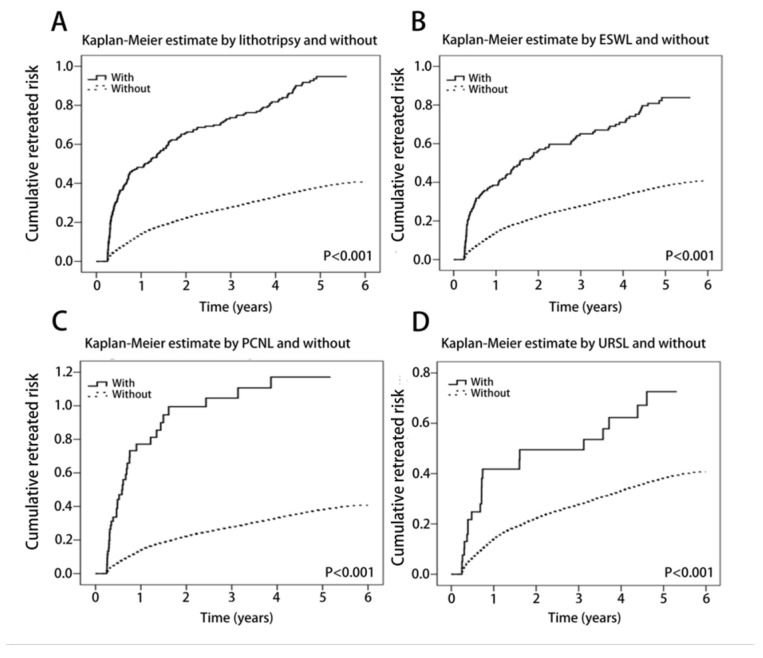
Kaplan–Meier curve for the cumulative rate of re-treatment (calculus of the kidney, ureter, and lower urinary tract/renal coli) with the log-rank test. (**A**) Stratified by lithotripsy (ESWL, PCNL, or URSL); (**B**) stratified by ESWL; (**C**) stratified by PCNL; and (**D**) stratified by URSL (log-rank *p* < 0.001).

All the procedures in the lithotripsy study cohort group were associated with a significantly higher rate of re-treatment than the procedures in the comparison cohort group (all were *p* < 0.001). The urolithiasis re-treatment rate was significantly higher in the lithotripsy group (ESWL, PCNL, or URSL) as compared with the non-lithotripsy group, based on the Kaplan–Meier survival curve (log-rank test *p* < 0.001) (Figure 2A–D). During the follow-up period, urolithiasis re-treatment occurred in 59.8% (204/341) of the lithotripsy group and in 31.5% (433/1375) of the non-lithotripsy group (Figure 1). Moreover, highly significant associations between the re-treatment rate and the mean age, CCI, season, and level of care were also observed (*p* < 0.001) (Table 2).

### 3.2. Multivariate Analysis of the Re-Treatment Rate of Urolithiasis

To examine the differences in the urolithiasis re-treatment rate following lithotripsy procedures, a multivariate analysis was performed in this study. The urolithiasis re-treatment rate was significantly higher in the lithotripsy group than in the non-lithotripsy group based on the Cox regression model (HR 1.710; 95% CI 1.427–2.048; *p* < 0.001) (Table 3). The HRs of urolithiasis re-treatment in the lithotripsy group were 0.99 for age, 1.36 for CCI, 8.36 for spring, 4.37 for summer, 2.21 for medical centers, and 2.00 for regional hospitals (Table 4), as compared with the non-lithotripsy group.

To investigate the repeated treatment rate of subgroups stratified by lithotripsy procedures, the seven study subgroups and the comparison cohort group without lithotripsy were compared. A Cox regression multivariate analysis revealed that the risk of repeated treatment was associated with the various modalities of lithotripsy for each 1-year decrease in age (adjusted HR: 0.99; 95% CI: 0.979–0.991; *p* < 0.001) and for each 1-point increase in the CCI score (adjusted HR: 1.36; 95% CI: 1.238–1.498; *p* < 0.001) (Table 4). The multivariate adjustment also suggested an increased risk of repeated treatment with ESWL only (adjusted HR: 1.60; 95% CI: 1.292–1.978; *p* <0.001) and PCNL only (adjusted HR: 2.32; 95% CI: 1.616–3.329; *p* < 0.001) (Figure 2B,C). Additionally, URSL only showed a non-significant association with an increase in repeated treatment (adjusted HR: 1.82; 95% CI: 1.166–2.831; *p* = 0.01). Nevertheless, the ESWL + PCNL, ESWL + PCNL, ESWL + URSL, and ESWL + URSL + PCNL combinations did not show significant associations with an increase in the repeated treatment rate (Table 4).

## 4. Discussion

In this study, we aimed to compare urolithiasis re-treatment rates in patients undergoing lithotripsy (ESWL, PCNL, or URSL) with those who had not undergone lithotripsy. All cases were recruited during the year 2005, then re-treated cases only counted after 90 days of initial surgery, and then followed up in the year 2010. As can be seen, high re-treatment rates significantly occurred when patients received all three procedures alone (*p* value 0.008~<0.001), especially ESWL only or PCNL only, when analyzed using the Cox regression multivariate analysis (Table 4). Similar results were also observed in the univariate analysis with a *p* value < 0.001 (Table 2). Regarding the other factors, it appears that age, CCI, season (particularly spring and summer), and level of care are important parameters correlated with re-treatment events, given that the p-values demonstrated significance for each of these factors (all *p* < 0.001) (Table 2).

First, we observed a high re-treatment rate for all types of lithotripsy procedures based on the univariate analysis (Table 2). We subsequently analyzed the data using a multivariate Cox regression model to examine the other parameters. The hazard ratio showed a high risk of re-treatment for patients who received ESWL only (HR: 1.60; 95% CI: 1.292–1.978; *p* < 0.001) or PCNL only (HR: 2.32; 95% CI: 1.616–3.329; *p* < 0.001) relative to those who received conservative observations (Table 3). Similar results were published showing that the overall re-treatment rate after ESWL varied between 6.7% and 41.8% after 3 months to 5 years [21]. Furthermore, when a single procedure was used, specifically ESWL only and PCNL only, both groups presented a significantly higher risk of re-treatment (*p* < 0.001) (Table 2). Notably, it can be concluded that a higher risk of re-treatment is usually observed in patients with more severe clinical conditions; in the present study CCI was analyzed related to urolithiasis [34,35]. In contrast, patients with a stone size less than 0.5 cm are likely to spontaneously pass the stones under surveillance if patients experience only minor symptoms. Additionally, factors related to the location, such as food, environment, and hospital care, can also differ across countries and should always be considered [35,36,37,38,39,40].

When comparing patients who received any one of the lithotripsy procedures, i.e., ESWL, PCNL, or URSL, to the patients who did not receive any surgery, the adjusted hazard ratio is 1.710 with a 95% CI 1.427–2.048). We then further combined all the treatment modalities of ESWL, PCNL, URSL, and the re-treatment rate was significantly higher than that observed in the matched comparison cohort during the entire follow-up period (*p* < 0.001) (Table 3).

One probable explanation for ESWL resulting in a higher risk of re-treatment is the location of the stone re-treatment in the ESWL group. When the stone shifts from the baseline location to the calyces before treatment, it might cause a higher re-treatment incidence within the calyces. We also know that calculi predominantly recurred in the lower calyx after ESWL (68%) [17,41]. Indeed, stone size, location, multiplicity, composition, a positive history of stone disease, and urinary tract infection after ESWL should all be considered relevant factors, and have been previously identified in other studies [24,41,42]. Unfortunately, information on stone size is missing from NHIRD’s database, so we were unable to assess this important factor. Previous studies failed to show a statistically significant difference in stone-free rates between ESWL and URS when treating small lower pole renal calculi [43,44]. However, ESWL was associated with greater patient acceptance and shorter convalescence [21,45]. Worcester et al. (2006) reported that urinary creatinine clearance data in cysteine stone, uric acid, calcium oxalate, apatite, and struvite stone formers had a much lower creatinine clearance than non-stone patients. Hyperuricosuria and low urinary pH (below 5.05) were also significant risk factors for uric acid crystallization and the development of stones [22,46]. Those renal-related factors that may predispose the kidney to new stone formation and/or crystal deposition were also risk factors for re-treatment. Nevertheless, our findings indicated that, as long as urolithiasis patients received ESWL (noted as the ESWL all group), the risk of the urolithiasis re-treatment significantly increased as compared with the risk for those who received other non-lithotripsy procedures (*p* = 0.001) (Table 3). This might be because of the presence of microscopic residual stone fragments left in the collecting system that may act as nidi for stone recurrence. Moreover, the exposure of the stone area to a lithogenic environment due to ESWL may also support new stone growth through heterogeneous nucleation and crystal aggregation [47].

Second, our data indicated that the incidence of repeated urolithiasis treatment after any lithotripsy procedure significantly decreases as age increases, as revealed by a Cox regression analysis (Table 1). Similar data were attained using a univariate analysis, and the highest age-specific repeated treatment incidence was observed in patients aged 18–30 years old (44%). Notably, patients over 65 years experienced a much lower re-treatment rate (27.1%) (Table 3) [48].

Third, CCI was also significantly correlated with the re-treatment rate in patients 90+ days after treatment. Comorbidity refers to one or more diseases co-occurring with the primary disease, and possibly related to another condition. Therefore, it is necessary to determine whether there is a predisposition to other diseases, or whether there is no link. Kidney stone recurrence sometimes contributes to poorer health outcomes, and therefore comorbid illness should be considered. In the current study, as long as the patients participated the disease associated with CCI would be analyzed [29,30,31]. Several diseases were considered in this study, including hypertension (ICD-9 cm codes 401~405), diabetes mellitus (ICD-9 cm code 250), heart failure (ICD-9 cm code 428), stroke (ICD-9 cm codes 430~438), chronic obstructive pulmonary disease (ICD-9 cm codes 490~496), liver cirrhosis (ICD-9 cm code 571), and Meniere’s disease (ICD-9 cm code 386.0). All these revealed an association with an increased risk of urolithiasis. Analyses with both univariate and multivariate Cox proportional hazards regression models showed a significant risk, with an HR of 1.36 and *p* < 0.001 (Table 2 and Table 3). It is well known that hypertension, diabetes mellitus, heart failure, stroke, and liver cirrhosis are all highly corelated with excessive body weight, and obesity is one of the factors of renal stone formation [34,36,37,38,39,40,49,50]. Indeed, for the other diseases, i.e., chronic kidney disease, hyperuricemia, cancer, urinary tract infections, hypercalcemia/hypercalciuria, etc., should be also taken into consideration, and they could be related to kidney stone formation; however, we only had limited authority access to the National Health Insurance Research Database in Taiwan, which is the limitation of present study. Moreover, seasonal variables, particularly spring and summer, were associated with a significantly higher risk of urolithiasis re-treatment. Both climatic and geographic factors are associated with urolithiasis, including temperature, season, sunshine hours, humidity, air pressure, and rainfall [51]. The prevalence of urolithiasis is higher in tropical and subtropical regions than in temperate and cold regions (5–10% vs. 1–5%) [22]. Hot and dry climates, typical of West Asia, can accelerate the evaporation of body water from the skin and consequently lead to urine concentration, a risk factor for crystal precipitation and stone formation. Similarly, the level of hospital care was relevant. In Taiwan, medical institutions are divided into three levels: “local hospitals”, “regional hospitals”, and “medical centers”. When people are sick, they can go to the local hospitals first. If the treatment is not as expected, more advanced examinations are needed, or the condition is unstable and the patient is hospitalized for care, the doctor will transfer them to other specialized local hospitals, or to a nearby larger regional hospital. Generally, the higher the medical level and the larger the scale, the more specialized the medical equipment and medical personnel. Therefore, medical centers and regional hospitals both showed higher frequencies of re-treatment cases than local hospitals. This phenomenon can be attributed to the high standard of medical care available in medical centers, which encourages patients with more serious clinical symptoms to seek a higher level of medical care.

Lastly, we discovered that the re-treatment rate was higher in male patients than in female patients. Therefore, it is necessary to address the varying factors among patients and devise appropriate individualized therapies to successfully prevent new stone formation and the subsequent growth of residual stones.

## 5. Conclusions

Regardless of the type of lithotripsy, a high risk of re-treatment existed for those who received ESWL and PCNL. The PCNL group, in particular, showed a highly significant association as compared with the other groups. Although the exact mechanisms of urolithiasis remain unclear, factors such as age, CCI, and season all contribute to recurrent stone formation. The selection of the most suitable lithotripsy procedure, however, always depends on the patients’ clinical conditions. Furthermore, every procedure has unique limitations. Our findings contribute to identifying high-risk patients in order to provide proper monitoring and investigation and to prevent future stone occurrence. We hope that the current results help in clinical decision-making.

## Figures and Tables

**Table 1:** Sex, age group, and level of care distributions of the lithotripsy study cohort group and the comparison cohort group.

Variables	Number of Individuals (%)	
	With Lithotripsy	Without Lithotripsy
	(n = 350)	(n = 1400)
*Sex*		
Male	216 (61.71%)	864 (61.71%)
Female	134 (38.29%)	536 (38.29%)
*Age (years, mean ± SD)*	50.34 ± 14.32	50.27 ± 14.54
*Age group (years)*			
18–30	28(8.00%)	112 (8.00%)
31–40	70 (20.00%)	280 (20.00%)
41–50	97 (27.71%)	388 (27.71%)
51–65	101 (28.86%)	404 (28.86%)
>65	54 (15.43%)	216 (15.43%)
*Level of care*			
Medical center	52 (14.86%)	208 (14.86%)
Regional hospital	95 (27.14%)	380 (27.14%)
Local hospital	203 (58.00%)	812 (58.00%)

**Table 2:** Univariate regression analysis of characteristics associated with re-treatment rate in urolithiasis patients with/without lithotripsy treatment.

Univariate Analysis											
Variables			Case Numbers n (%)		With Re-Treatment n (%)			Without Re-Treatment n (%)			*p-*Value
*Total number and percentage*				1716	(100.00)	637	(37.12)		1079	(62.88)	
Lithotripsy *											<0.001
		With	341	(19.87)	204	(32.03)		137	(12.70)		
		Without *	1375	(80.13)	433	(67.97)		942	(87.30)		
	ESWL										<0.001
		With	241	(14.04)	142	(22.29)		99	(9.18)		
		Without	1475	(85.96)	495	(77.71)		980	(90.82)		
	PCNL										<0.001
		With	79	(4.60)	59	(9.26)		20	(1.85)		
		Without	1637	(95.40)	578	(90.74)		1059	(98.15)		
	URSL										<0.001
		With	53	(3.09)	32	(5.02)		21	(1.95)		
		Without	1663	(96.91)	605	(94.98)		1058	(98.05)		
*Lithotripsy*											<0.001
	Without		1375	(80.13)	433	(67.97)		942	(87.30)		
	ESWL only		214	(12.47)	118	(18.52)		96	(8.90)		
	PCNL only		56	(3.26)	38	(5.97)		18	(1.67)		
	URSL only		41	(2.39)	21	(3.30)		20	(1.85)		
	ESWL + PCNL		18	(1.05)	16	(2.51)		2	(0.19)		
	ESWL + URSL		7	(0.41)	6	(0.94)		1	(0.09)		
	PCNL + URSL		3	(0.17)	3	(0.47)		0	(0)		
	ESWL + PCNL + URSL			2	(0.12)	2	(0.31)		0	(0)	
**Sociodemography**											
*Sex*											0.01
	Male		1057	(61.60)	418	(65.62)		639	(59.22)		
	Female		659	(38.40)	219	(34.38)		440	(40.78)		
*Age (mean ± SD)*			53.90 ± 14.36		52.07 ± 13.72			54.97 ± 14.63			<0.001
*Age group (years)*											0.01
	18–30		75	(4.37)	33	(5.18)		42	(3.89)		
	31–40		250	(14.57)	103	(16.17)		147	(13.62)		
	41–50		458	(26.69)	175	(27.47)		283	(26.23)		
	51–65		572	(33.33)	228	(35.79)		344	(31.88)		
	>65		361	(21.04)	98	(15.38)		263	(24.37)		
*CCI (mean ± SD)*			0.04 ± 0.44		0.11 ± 0.72			0.01 ± 0.01			<0.001
**Environmental factors**											
*Season*											<0.001
	Spring (March–May)			301	(17.54)	237	(37.21)		64	(5.93)	
	Summer (June–August)			238	(13.87)	151	(23.70)		87	(8.06)	
	Autumn (September–November)			444	(25.87)	116	(18.21)		328	(30.40)	
	Winter (December–February)			733	(42.72)	133	(20.88)		600	(55.61)	
**Hospital-related**											
*Level of care*											<0.001
	Medical center		214	(12.47)	115	(18.05)		99	(9.18)		
	Regional hospital		385	(22.44)	214	(33.59)		171	(15.85)		
	Local hospital		1117	(65.09)	308	(48.35)		809	(74.98)		

Nine and twenty-five individuals were excluded from the study cohort group and the comparison cohort group, respectively. Lithotripsy *: included ESWL, PCNL, or URSL. Without *: control group. ESWL: extracorporeal shockwave lithotripsy; PCNL: percutaneous nephrolithotomy; URSL: ureteroscopic lithotripsy; CCI: Charlson comorbidities index. *p-*value was < 0.05. Bold means differ categories.

**Table 3:** Multivariate analysis of the characteristics of patients involved with/without lithotripsy study.

			Multivariate Analysis			
Variables			Adj. HR *	95% CI	95% CI	*p-*Value
*Lithotripsy (ESWL, PCNL, or URSL)*						
	With		1.710	1.427	2.048	**<0.001**
	Without		Reference			

The dependent variable was repeated treatment (with/without), and *p* < 0.05 was significant. * Adj. HR is represented as the adjusted hazard ratio.

**Table 4:** Predictive ability regarding the independent factors of repeated treatment after lithotripsy analyzed by Cox regression multivariate analysis.

Variables		Multivariate Analysis		
		Adj. HR *	95% CI	*p-*Value
*Total number and percentage*				
*Lithotripsy*				
	Without	Control		
	ESWL only	1.60	1.292–1.978	**<0.001**
	PCNL only	2.32	1.616–3.329	**<0.001**
	URSL only	1.82	1.166–2.831	**0.008**
	ESWL + PCNL	0.99	0.520–1.900	0.986
	ESWL + URSL	2.54	1.107–5.817	**0.028**
	PCNL + URSL	1.26	0.398–3.986	0.695
	ESWL + PCNL + URSL	6.68	1.633–27.325	**0.008**
Socio-demography				
*Sex*				
	Male	1.02	0.855–1.215	0.833
	Female	Control		
*Age (mean ± SD)*		0.99	0.979–0.991	**<0.001**
*CCI (mean ± SD)*		1.36	1.238–1.498	**<0.001**
Environmental factors				
*Season*				
	Spring (March–May)	8.36	6.628–10.556	**<0.001**
	Summer (June–August)	4.37	3.414–5.589	**<0.001**
	Autumn (September–November)	1.37	1.059–1.760	**0.016**
	Winter (December–February)	Control		
Hospital-related				
*Level of care*				
	Medical center	2.21	1.736–2.816	**<0.001**
	Regional hospital	2.00	1.633–2.368	**<0.001**
	Local hospital	Control		

Nine and twenty-five individuals were excluded from the study cohort group and the comparison cohort group, respectively; * Adj. HR is represented as the adjusted hazard ratio; ESWL: extracorporeal shock wave lithotripsy; PCNL: percutaneous nephrolithotomy; URSL: ureteroscopic lithotripsy; CCI: Charlson comorbidities index; *p* < 0.05 was considered significant.

## Data Availability

Data were obtained from a third party and are not publicly available.
